# Surrogacy, an Excellent Opportunity for Women with More Threats

**Published:** 2019

**Authors:** Mohammad Reza Sadeghi

Surrogacy is one of the assisted reproduction methods which have a very long history. The first traditional surrogacy was performed in about 2000 years before the birth of Christ as mentioned in the Holy Qur’an of Muslims and the Christians Bible; Sarah was the wife of Abraham prophet and she was sterile which made her unable to conceive. Hajar was a female servant of Sarah who asked to carry a child for Abraham prophet. Afterward, Hajar delivered a son for Sarah named Ishmael. This is the traditional, genetic or partial form of surrogacy; it has already been performed by artificial insemination of the surrogate with sperm of intended father and the usage of this form of surrogacy has been greatly reduced. This surrogate mother not only carries the embryo till full term delivery but also provides the eggs that make her a genetic parent. Moreover, the most popular form is gestational, or IVF surrogacy in which an embryo from the intended parents, or from a donated oocyte or sperm, is transferred to the surrogate’s uterus. In gestational surrogacy, the mother who carries and delivers the child (the gestational carrier) has no genetic relation with the child (1).

Surrogacy is used for approximately 2% of embryo transfer cycles in USA. The indications for surrogacy include absence of the uterus, recurrent pregnancy loss, repeated IVF failure, poor obstetric history, contraindication of pregnancy, excessive maternal risk, etc. Surrogacy improves pregnancy and live birth rate compared to other

ART cycles without surrogacy. The best results are observed in surrogate candidate due to uterine factor infertility (2).

Most of the studies reported better prenatal outcomes in surrogacy including hypertension, preeclampsia, gestational diabetes, placenta previa, preterm labor and lower birth weight in comparison to IVF cycles of their own uterus. However, when these adverse outcomes in surrogate mothers were compared with the same surrogate mothers who became pregnant through spontaneous conception, IVF manifested most of the above adverse outcomes due to laboratory manipulation of gametes and embryos and uterine milieu changes in stimulation cycles (3).

The surrogacy needs IVF facilities without considering infertility etiology, so gestational carriers experienced embryo transfer, pregnancy, successful ongoing pregnancy and delivery. Therefore, precise medical, psychological and social assessments are necessary to succeed in gestation and delivery. Health status of carriers during gestation could affect future health and wellbeing of the child. The history profile of surrogate should contain at least one uncomplicated pregnancy, although not more than 5 deliveries or 3 caesarean sections (4).

In spite of most benefits of surrogacy and also considering the fact that it may be the only option for many couples to have a child from their own gametes, surrogacy is prohibited in many European countries such as Germany, Sweden, Norway, and Italy. Due to financial payments and maintaining the dignity and rights of individuals, any payment to surrogate is officially forbidden in many other countries and only compensation of pregnancy-related costs is accepted in Australia, UK, and in many states of USA. Commercial surrogacy is accepted in India, Ukraine, USA and Middle East countries. One of the remarkable cases is accompaniment of surrogacy with oocyte donation cycles, so that 46% of surrogacy cycles in the USA involve donor eggs. Oocyte donation by carrier for intended parents is prohibited in almost all countries (4).

All of these strict regulations have been set up to protect the rights of the parties involved in this sensitive and complex process, including child, surrogate and intended parents. Inappropriate practice of surrogacy in cases without medical indication such as celebrities, businesswomen and female politicians for the birth of their child in developed countries is another concern of the field. Cross-border commercial surrogacy in poor developing countries such as India, Nepal, Thailand, and Mexico is another concern. Therefore, in spite of the importance and critical role of surrogacy in assisted reproduction technologies and its existence as the only option for many couples in childbearing age, it requires more consideration and more strict regulation at global level compared to current status due to misuse of the practice in unnecessary cases.
